# A single-center case-control study of the association between dorsal striatal damage and nicotine addiction

**DOI:** 10.3389/fneur.2025.1553200

**Published:** 2025-05-19

**Authors:** Chuya Jing, Xingkai An, Jie Fang

**Affiliations:** ^1^Department of Neurology, The First Affiliated Hospital of Xiamen University, School of Medicine, Xiamen University, Xiamen, China; ^2^Department of Neuroscience, The First Affiliated Hospital of Xiamen University, School of Medicine, Xiamen University, Xiamen, China; ^3^Fujian Key Laboratory of Brain Tumors Diagnosis and Precision Treatment, Xiamen, China; ^4^Xiamen Key Laboratory of Brain Center, Xiamen, China; ^5^Xiamen Medical Quality Control Center for Neurology, Xiamen, China; ^6^Fujian Provincial Clinical Research Center for Brain Diseases, Fuzhou, China; ^7^Xiamen Clinical Research Center for Neurological Diseases, Xiamen, China; ^8^The School of Clinical Medicine, Fujian Medical University, Fuzhou, China; ^9^Fujian University of Traditional Chinese Medicine, Fuzhou, China

**Keywords:** smoking, nicotine addiction, dorsal striatum, damage, cessation

## Abstract

**Background:**

Smoking is closely linked to pulmonary diseases, cardiovascular diseases, cancer, and other health issues, posing a significant threat to human health. The essence of smoking addiction lies in nicotine dependence, though the underlying mechanisms remain unclear. Previous studies have suggested that nicotine addiction is associated with regions such as the insula, globus pallidus, amygdala, and ventral striatum. However, our prior retrospective study indicates that the dorsal striatum may also play a role in nicotine addiction.

**Objective:**

Our research aims to evaluate the alterations in nicotine addiction among patients with dorsal striatum lesions at 1 month, 3 months, 6 months, and 12 months post-injury.

**Methods:**

We collected data from patients of the First Affiliated Hospital of Xiamen University between August 2021 and August 2024. We screened some patients with dorsal striatum injury and asked a series of questions at the time points of 1 month, 3 months, 6 months, and 12 months after brain injury. Ultimately, 13 patients with dorsal lesions who met the inclusion criteria and a control group of 13 patients with brain injuries in other regions were selected.

**Results:**

We observed that patients with dorsal striatum damage experienced smoking cessation more readily and earlier compared to the control group. Furthermore, those with more severe dorsal striatum damage might maintain this cessation for longer. Additionally, nicotine dependence scores on the Fagerström Test for Nicotine Dependence (FTND) were lower in patients with dorsal striatum damage compared to the control group, indicating a reduced level of nicotine dependence.

**Conclusion:**

This conclusion suggests that the dorsal striatum may be associated with nicotine addiction.

## Introduction

Nicotine addiction has emerged as a major public health concern (1), with over 7 million individuals worldwide succumbing to direct smoking-related causes annually (2). Smoking is closely related to various cardiovascular diseases, respiratory diseases, digestive diseases, tumors, and other illnesses (3, 4). Despite the increasing awareness of smoking’s detrimental health effects due to widespread health education, quitting smoking remains a formidable challenge. Smokers often experience a range of withdrawal symptoms, including physical, emotional, and cognitive negative effects, when attempting to quit (5). Data indicates that the relapse rate among smokers after cessation exceeds 85% (6).

Nicotine, a primary psychoactive alkaloid found in tobacco plants, is a highly addictive substance (7). Extensive research has confirmed that the essence of smoking addiction is nicotine dependence. Similar to other substance addictions, nicotine addiction manifests as a chronic mental disorder characterized by compulsive smoking behavior (8). Upon inhalation of cigarette smoke, nicotine is rapidly absorbed through the oral, nasal, and bronchial mucosa into the bloodstream, subsequently crossing the blood-brain barrier to enter the central nervous system, inducing immediate pleasurable effects and anxiety relief. The data from animal studies exhibit that animals show a series of changes at the cellular and molecular levels within the mesolimbic dopamine system after prolonged and repeated exposure to nicotine, leading to neuroplasticity alterations and resulting in compulsive drug use and nicotine addiction (9).

The molecular mechanisms underlying nicotine addiction remain unclear. Previous studies suggest that numerous brain regions are involved in addiction pathways, including the insula, globus pallidus, amygdala, nucleus accumbens, and ventral striatum (2, 10–12). Research on the role of the striatum in nicotine addiction has predominantly focused on the ventral striatum (13–15). However, some studies have found that the dorsal striatum, crucial for goal-directed behavior and habit formation, also undergoes neuroplastic changes due to repeated nicotine exposure (16, 17). In one study, 18 adult smokers underwent fMRI scans under two conditions: regular smoking and 24-h abstinence. The results showed that, compared to the control group, the dorsal striatum was more active in participants after 24 h of abstinence, suggesting its involvement in the addiction process (18). Our previous retrospective study found that patients with dorsal striatum damage could quit smoking more easily and quickly without relapse and experienced reduced smoking urges (19). This indicates the need for further research into the role of the dorsal striatum in nicotine addiction.

## Materials and methods

### Subjects

All research participants were recruited from the First Affiliated Hospital of Xiamen University between August 2021 and August 2024. All procedures were supervised and reviewed by the First Affiliated Hospital of Xiamen University, and all subjects signed informed consent forms before participating in the study. We examined the patient’s electronic medical records. We conducted interviews to ensure they met the following inclusion criteria: (1) male patients with acute brain injury, with the injury localized solely to the dorsal striatum region; (2) patients who completed cranial MR examinations, with lesions observable on MRI; (3) patients with a history of smoking and a Fagerström Test for Nicotine Dependence (FTND) score of ≥7, indicating severe nicotine dependence; (4) patients without a significant history of psychiatric disorders or aphasia; (5) patients not addicted to substances other than nicotine; (6) patients were excluded if they developed new intracranial lesions or other serious illnesses during follow-up within 1 year of onset, or if they were confined to environments where smoking was prohibited.

### Subject selection

**Figure 1:**
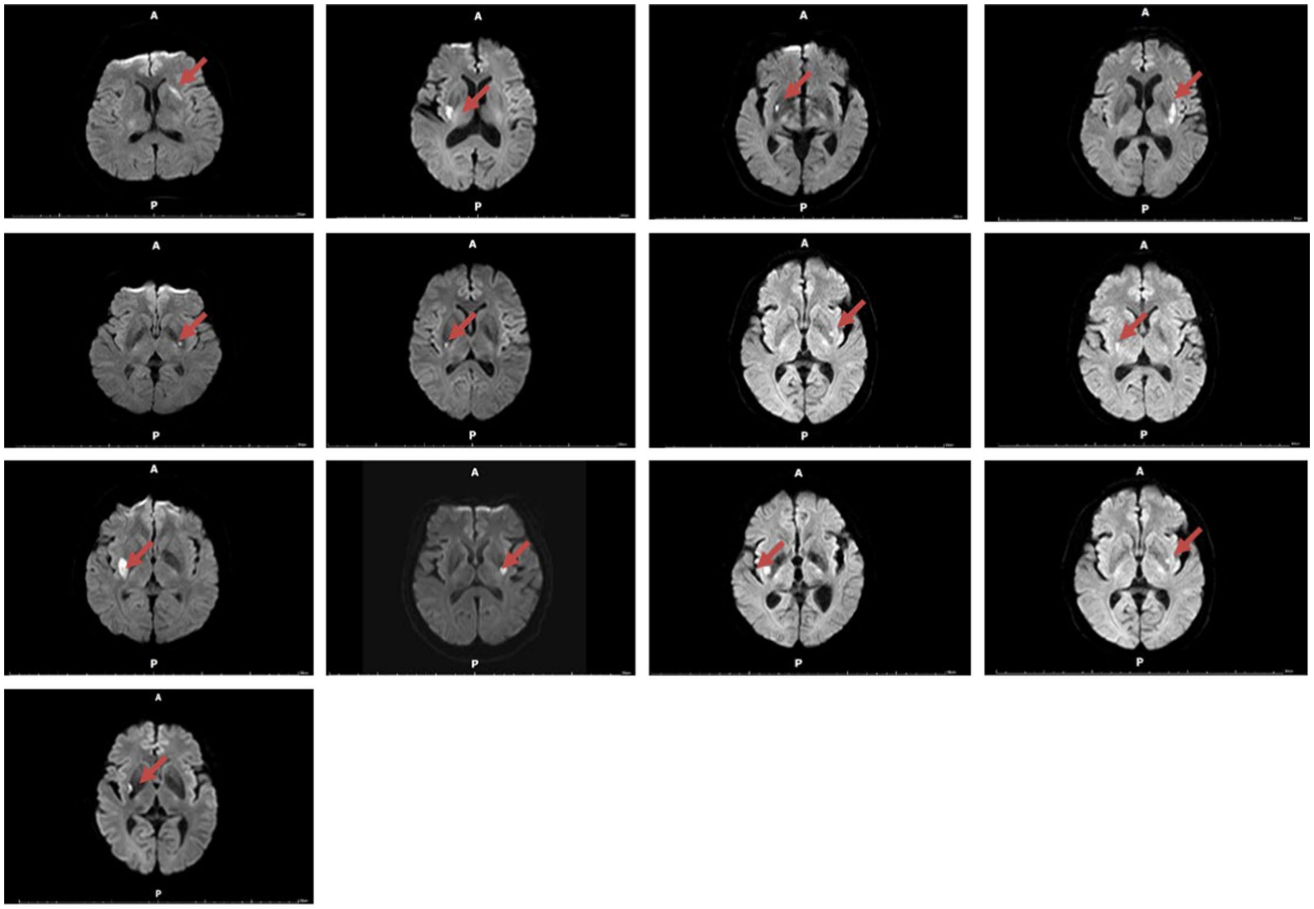
Magnetic resonance imaging (diffusion-weighted imaging, DWI) was performed on 13 smokers with dorsal striatum damage.

Between 2021 and 2024, 13 patients met our inclusion criteria (Figure 1). Additionally, we identified and screened 13 male patients who met conditions (2), (3), (4), (5), and (6) as the control group. The intracranial injuries in the control group were as follows: cerebellum (1 person), basal ganglia (3 persons), thalamus (2 persons), brainstem (3 persons), frontal lobe (1 person), insula (1 person), and corona radiata (2 persons). The cause of brain damage in all 26 patients was cerebral infarction. These 26 patients were ultimately included as subjects in this study and were incorporated into the statistical analysis.

### Measures

We obtained relevant information about the subjects by reviewing electronic medical records and conducting face-to-face, phone, and video interviews. This information included their educational background, employment status, living conditions, age of onset, years of smoking, daily cigarette consumption, FTND scores, and nicotine addiction status. The location of brain injury in all patients was determined jointly by a neurologist and two radiologists.

### Statistical analysis

Firstly, descriptive statistics were used to describe the basic demographic characteristics and baseline FTND scores of the dorsal striatum group and the non-dorsal striatum group. *t*-tests and Fisher’s exact tests were used for between-group comparisons. Second, bar charts were used to describe changes in smoking cessation among the study participants. Univariate logistic regression analysis was used to compare the number of quitters with disrupted smoking addiction in different groups at different time points. Odds ratios (ORs), 95% confidence intervals (CIs), and *p*-values were reported, with *p*-values adjusted using the Bonferroni method (the adjusted *p*-value in this study was 0.0125). Third, we compared the differences in FTND scores between the two groups at different time points, reported Cohen’s *d* and *p*-values, and adjusted *p*-values using the Bonferroni method. Fourth, repeated measures analysis of variance was used to compare the trends in FTND scores between the two groups (dorsal striatum and non-dorsal striatum) at different time points, and to evaluate the interaction between time and group. All significance tests were two-tailed, with *p* < 0.05. Data organization and statistical analyses were performed using R software version 4.4.1.

### Behavioral classification

We conducted interviews with 26 patients to assess changes in their smoking behavior at various intervals following the onset of their condition. Initially, we planned to interview patients from the first day of onset. However, considering potential interference from factors such as the patient’s mood, hospital environment, and treatment, we conducted interviews at four specific intervals: the first month, the third month, the sixth month, and the twelfth-month post-onset.

All patients were asked whether they had resumed smoking at any point after the onset. Those who reported smoking, even a single cigarette, were classified as “non-quitters,” while those who did not smoke were classified as “quitters” We refined Naqvi’s et al. (11) classification method by further questioning the “quitters” with the following inquiries: “How soon after your brain injury did you quit smoking?” “How difficult was it to quit smoking after the brain injury (rated on a scale of 1–7, with 1 being very easy and 7 being very difficult, based on the patient’s subjective experience)?” “Have you experienced any urges to smoke again after quitting?” Among the “quitters,” those who quit smoking within less than a day after the brain injury rated the difficulty of quitting as less than 3, and did not experience any urges to smoke again, were categorized as “disruption of smoking addiction.” The remaining patients were classified as having “no disruption of smoking addiction.” To prevent redundant and unnecessary inquiries, interviews at the 3-month, 6-month, and 12-month intervals will include selected questions based on responses from the initial 1-month interview.

### MRI acquisition

A 3.0 T MRI system (Ingenia, Philips Medical Systems, Netherlands) was used for all data collection. The head coil had a 16-channel phased-array. Diffusion-weighted imaging sequence with *b*-values = 1,000 s/mm^2^ (Time of Repetition = 1,407 ms; Time of Echo = 90 ms; Slices = 21; Thickness = 6 mm; gap = 1 mm; Flip Angle = 90°; Field of View = 224 × 224 mm; Matrix = 112 × 110; Voxel Size = 2 × 2.04 × 6.0 mm. Number of Signals Averaged = 1. The sequence took 1 min and 8 s).

## Results

We compared the baseline characteristics of 13 patients in the non-dorsal striatum group with 13 patients in the dorsal striatum group and found no significant differences in education level, employment status, living conditions, age of onset, years of smoking, daily cigarette consumption, and pre-onset FTND scores (*p* > 0.05) (Table 1).

**Table 1:** Characteristics of the dorsal and non-dorsal striatum groups.

Basic information	Overall *N* = 26^a^	Dorsal striatum *N* = 13^a^	Non-dorsal striatum *N* = 13^b^	*p*-value^b^
Highest education completed				0.674
≤Primary school	10 (38.46%)	5 (38.46%)	5 (38.46%)	
≤High school	12 (46.15%)	5 (38.46%)	7 (53.85%)	
>High school	4 (15.38%)	3 (23.08%)	1 (7.69%)	
Employment status				0.673
Hired	8 (30.77%)	5 (38.46%)	3 (23.08%)	
Workless	18 (69.23%)	8 (61.54%)	10 (76.92%)	
Living conditions				—
Living with relatives	26 (100%)	13 (100%)	13 (100%)	
Living alone	0	0	0	
Age at lesion onset, age	60.92 ± 13.75	61.23 ± 14.36	60.62 ± 13.70	0.912
Years of smoking at lesion onset	32.31 ± 12.59	33.46 ± 13.29	31.15 ± 12.27	0.650
Cigarettes smoked per day at lesion onset	20.23 ± 7.33	18.85 ± 5.06	21.62 ± 9.07	0.349
FTND score	7.35 ± 0.49	7.31 ± 0.48	7.38 ± 0.51	0.695

a *n* (%); mean ± SD.

b Fisher’s exact test; Welch two sample *t*-test.

**Figure 2:**
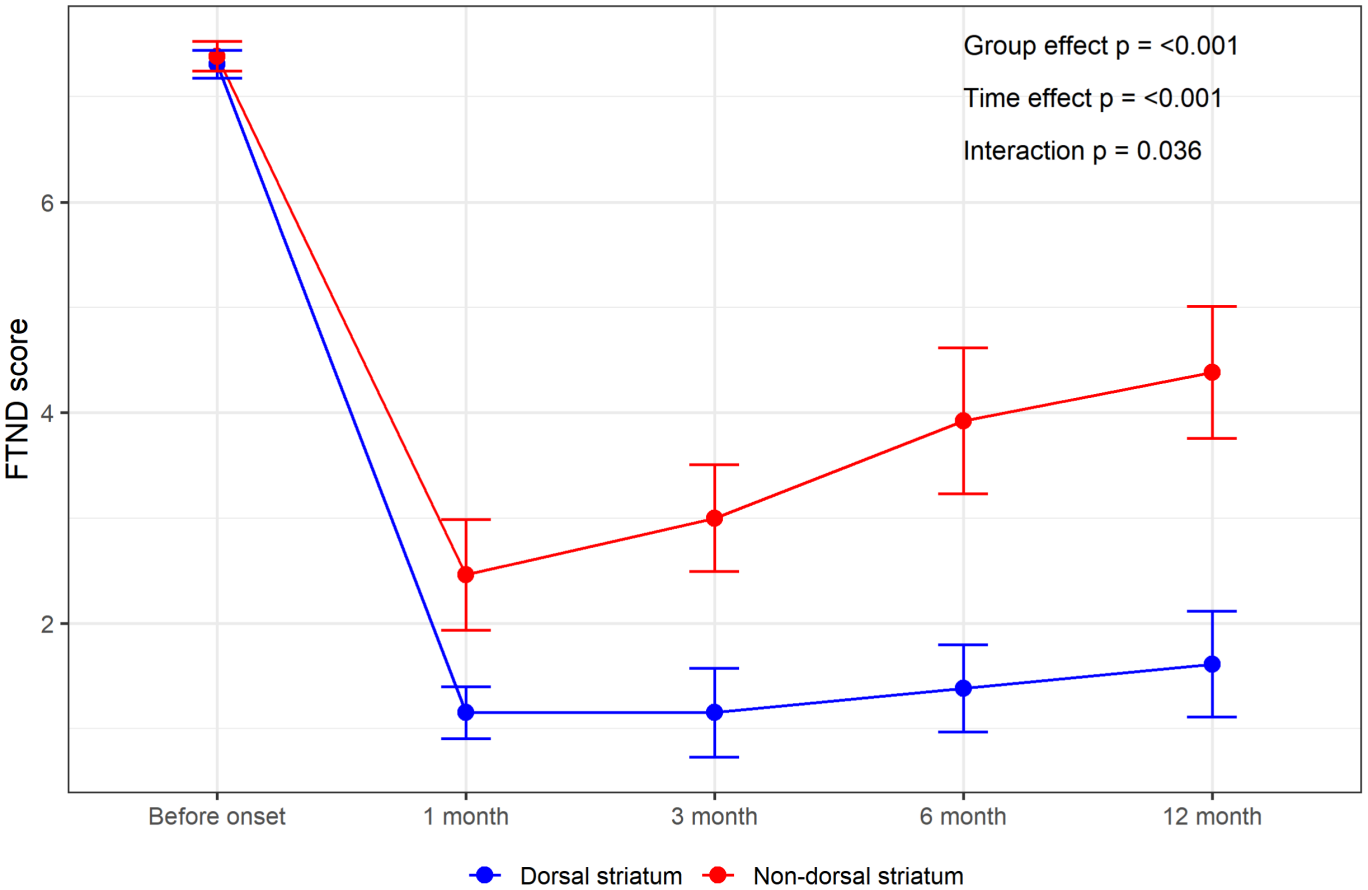
The line chart shows the average FTND scores of the dorsal and non-dorsal striatum groups at different times.

Based on the description in the behavioral classification section, we surveyed two groups of patients at four time points: 1 month, 3 months, 6 months, and 12 months after onset, to understand their smoking addiction status. Our study observed that patients with dorsal striatum damage have a significantly higher likelihood of experiencing smoking addiction interruption at 1 month, 3 months, 6 months, and 12 months post-onset compared to the non-dorsal striatum damage group, with statistically significant differences (Table 2). Additionally, we analyzed the changes in FTND scores over different periods for both groups. Although there was no significant difference in FTND scores between the two groups before onset (*p* = 0.695), the average FTND scores for patients with dorsal striatum damage were significantly lower than those of the control group at each time point post-onset (Figure 2). There was a significant difference in the overall scores between the two groups (group effect, *p* < 0.001), and the scores changed significantly over time (time effect, *p* < 0.001). The interaction effect was also significant (*p* = 0.036), indicating that the two groups had different patterns of score changes, with the dorsal striatum group’s FTND decreasing and then trends stabilizing (Table 3).

**Table 2:** Number of quitters with disruption of smoking addiction in different groups at different times.

Follow-up time	Dorsal striatum *N* = 13^a^	Non-dorsal striatum *N* = 13^a^	OR^a^	95% CI^a^	*p*-value^b^
1 month	11 (84.62%)	2 (15.58%)	30.25	4.433, 353.6	0.002
3 months	11 (84.62%)	2 (15.58%)	30.25	4.433, 353.6	0.002
6 months	10 (76.92%)	2 (15.58%)	18.33	2.985, 175.9	0.004
12 months	10 (76.92%)	2 (15.58%)	18.33	2.985, 175.9	0.004

a OR, odds ratio; CI, confidence interval.

b The logistic regression analysis revealed that all *p*-values were below the Bonferroni-adjusted threshold (0.05/4 = 0.0125), suggesting that the observed differences are statistically significant.

**Table 3:** The average FTND scores of different groups and periods.

FTND score	Overall *N* = 26^a^	Dorsal striatum *N* = 13^a^	Non-dorsal striatum *N* = 13^a^	Cohen *d*	*p*-value^b^
FTND score (1 month)	1.81 ± 1.60	1.15 ± 0.90	2.46 ± 1.90	0.88 (0.03, 1.73)	0.038
FTND score (3 months)	2.08 ± 1.90	1.15 ± 1.52	3.00 ± 1.83	1.10 (0.23, 1.97)	0.010
FTND score (6 months)	2.65 ± 2.40	1.38 ± 1.50	3.92 ± 2.50	1.23 (0.35, 2.11)	0.005
FTND score (12 months)	3.00 ± 2.45	1.62 ± 1.80	4.38 ± 2.26	1.36 (0.46, 2.25)	0,002

a Mean ± SD.

b Welch two sample *t*-test. All *p*-values were below the Bonferroni-adjusted threshold (0.05/4 = 0.0125), suggesting that the observed differences are statistically significant.

Furthermore, we discovered that among the dorsal striatum damage group, one patient consistently smoked from the onset, one patient consistently classified as “no disruption of smoking addiction,” and the third patient was classified as having “disruption of smoking addiction.” At the 1-month and 3-month follow-ups but exhibited smoking impulses at 6 months, classified as “no disruption of smoking addiction,” and had resumed smoking by the 12-month interview.

**Figure 3:**
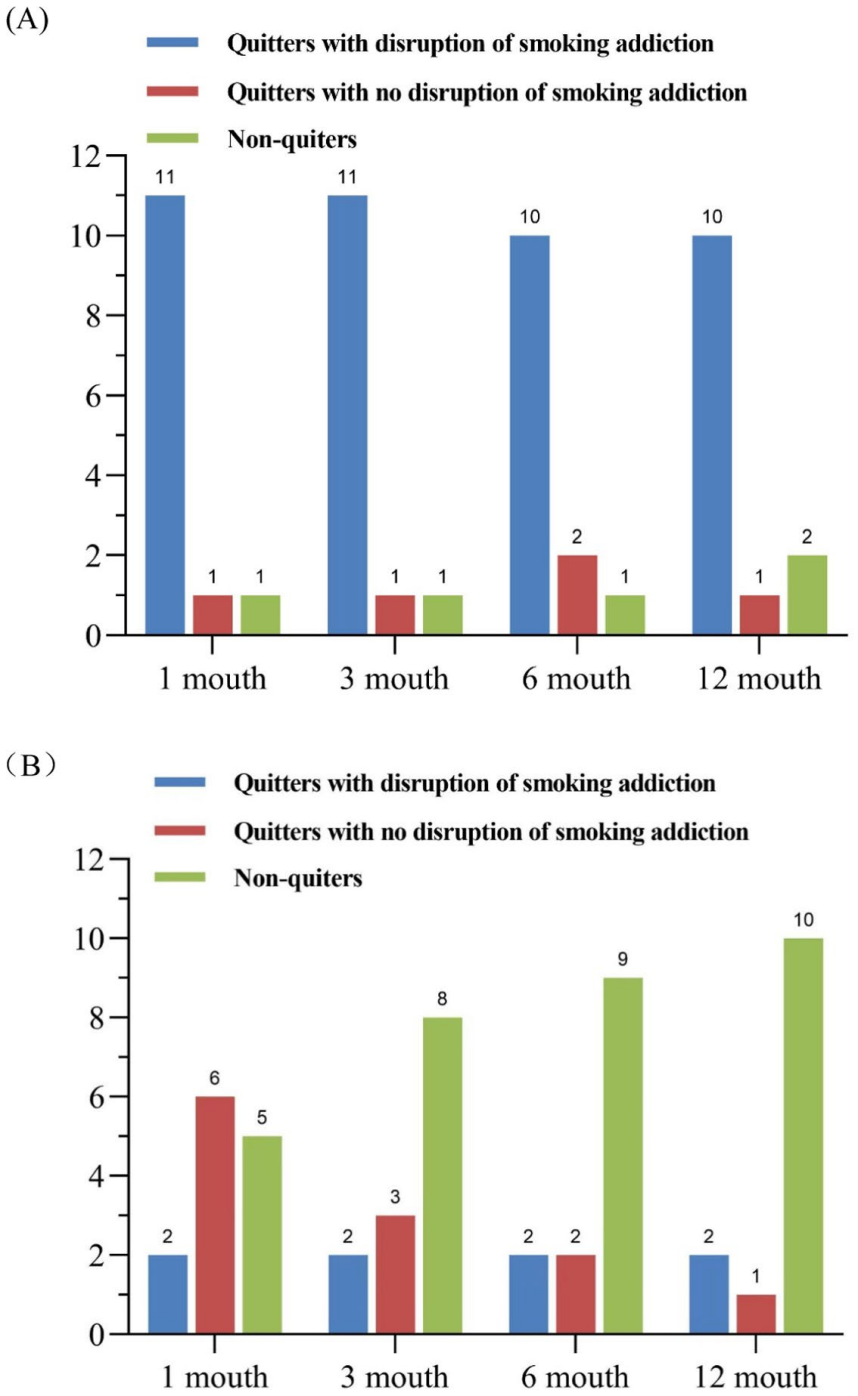
**(A)** The bar chart shows the addiction status of patients in the dorsal striatum group at different times. **(B)** The bar chart shows the addiction status of patients in the non-dorsal striatum group at different times.

In the non-dorsal striatum damage group, two patients exhibited “disruption of smoking addiction” immediately after illness, with damage located in the insula and frontal lobe, respectively. This may be because the insula and cortex may also be involved in the smoking addiction process, as previous reports have confirmed their potential involvement in addiction. Some other patients, although not immediately exhibiting smoking behavior post-onset and classified as having “no disruption of smoking addiction” in behavioral classification, found it difficult to maintain this status and resumed smoking relatively early. More details are shown in Figures 3A,B.

## Discussion

Our research has clinically observed that patients with dorsal striatal lesions are more likely to quit smoking, characterized by faster and easier cessation, longer abstinence, and lower relapse rates. In this study, three patients with dorsal striatal lesions were not classified as having “disruption of smoking addiction” at the 12-month follow-up. The common feature of these three patients is that the lesions in the dorsal striatum are relatively small. We speculate that the nicotine dependence of these three patients may be because the dorsal striatal lesions were small, and their function was not significantly altered. Of course. Another explanation is that because the patient’s lesions were mild and their clinical symptoms recovered quickly, they did not believe psychologically that smoking would cause serious consequences and thus did not quit smoking or resumed smoking. In addition, compared with the control group, patients with dorsal striatal damage had lower FTND scores at different time points, indicating lower nicotine dependence. During the follow-up process, one patient described that after dorsal striatal damage, in addition to quitting smoking, he felt that his anxiety symptoms had decreased significantly and his mood was more stable. We speculate that this is related to changes in dopamine secretion in the brain. In addition, some patients reported that after quitting smoking, they had improved physical fitness, were more focused at work, and felt more confident in social situations.

One thing that must be emphasized is that it is still unclear which areas of the brain are involved in the process of nicotine addiction, so we cannot completely avoid the regions involved in nicotine addiction when setting up the control group. In this study, there were one patient with an insular lobe injury and one with a frontal lobe injury who were classified as having a “disruption of smoking addiction” after the onset of the disease. As mentioned earlier, some studies have found that the insula is involved in addiction, while the frontal lobe and dorsal striatum have fiber connections (11, 12). These potentially involved regions in the control group may affect the results.

Previous studies have indicated that substance abuse can lead to increased sensitivity of the neural reward circuitry and impaired inhibitory control, resulting in the overactivation of the reward pathway, which is considered one of the key mechanisms in addiction formation. Within the reward circuitry, the mesolimbic dopamine system (MLDS) plays a significant role. The MLDS comprises several regions, including the ventral tegmental area (VTA), nucleus accumbens (NAc), hippocampus, amygdala, and prefrontal cortex. The MLDS serves as the structural basis for reward effects and plays a crucial role in the rewarding effects of addictive substances (20).

In the nervous system, the widely expressed nicotinic acetylcholine receptors (nAChRs), which belong to the ligand-gated ion channel family, mediate rapid synaptic transmission by altering cation channel flux. Some studies suggest that nicotine from tobacco can cross the blood-brain barrier and bind to nAChRs, activating different subtypes of nAChRs on various cell populations within the VTA of the MLDS. This activation stimulates dopaminergic neurons in the VTA, which then project to the NAc, promoting dopamine release in the NAc and producing rewarding effects (21). NAc, a key structure in the ventral striatum, is closely related to smoking motivation, anticipated pleasure, and withdrawal symptoms, and is thought to play a crucial role in nicotine relapse (22).

The striatum is divided into the ventral striatum (VS) and dorsal striatum (DS) based on structural and functional differences. The ventral striatum includes brain regions such as the nucleus accumbens and olfactory tubercle, while the dorsal striatum comprises the dorsomedial striatum (DMS) and dorsolateral striatum (DLS). Within the dorsal striatum, there are γ-aminobutyric acidergic (GABA) interneurons, cholinergic interneurons, and somatostatin-expressing (SOM) interneurons, all of which play crucial roles in local neural circuits (23, 24).

Previous studies on the role of the dorsal striatum in nicotine addiction are scarce, with even fewer focusing on molecular mechanisms. Some studies suggest that the striatum is involved in the later stages of addiction and that there appears to be a neuroanatomical progression from the ventral striatum to the dorsal striatum during this stage. Research on drugs has found that the behavior of seeking drugs originates in the dorsal striatum (25). A recent electrophysiological recording in an animal experiment observed a selective increase in excitatory neurotransmitters in the dorsal striatum during acute withdrawal (26). Based on the current study, we hypothesize that the dorsal striatum is linked to nicotine addiction through the following mechanisms:

### Dopamine mechanism

Numerous studies have shown that nicotine intake during smoking can lead to dopamine release (27, 28). Previous studies have found that certain addictive drugs (such as cocaine) can induce dopamine release in the dorsal striatum (28–30) and that this increase in dopamine release may be achieved through changes in the astrocytes of the dorsal striatum (31). In a recent animal study, it was found that caffeine, nicotine, and ethanol, when injected into the brains of rats, can significantly increase the release of dopamine in the dorsal striatum (32). Changes in dopaminergic neurotransmission in the dorsal striatum are further associated with drug abuse, compulsive drug-seeking behavior, craving, and relapse, which are critical components of the later stages of addiction. Therefore, we speculate that one possible mechanism for addiction cessation associated with dorsal striatum damage is the reduced nicotine-induced dopamine release in the dorsal striatum, thereby affecting this addiction mechanism.

### Glutamate mechanism

While dopamine plays a crucial role in acute drug exposure, neuroadaptive changes in the glutamate system may be vital for the development of compulsive drug-seeking behavior (33). Increasing preclinical evidence suggests that cocaine-induced alterations in neurotransmitters, including glutamate and γ-aminobutyric acid (34, 35), may underlie the formation of maladaptive habits (36). Studies indicate that the dorsal striatum receives glutamatergic inputs from upstream cortical and thalamic regions, acting as a relay station that integrates upstream information and transmits signals to downstream nuclei via direct and indirect pathways (37). A recent animal experiment confirmed that the interaction between the activated glutamatergic projections from the orbitofrontal cortex and dopamine 1-family receptor expression in dorsal striatum was activated during incubated oxycodone seeking (38). NMDA receptors, a type of glutamate receptor, are involved in habitual learning in the dorsolateral striatum of rats (39, 40), with behavioral training enhancing glutamatergic neurotransmission in this area (41). Drug-induced changes in glutamatergic input to the dorsolateral striatum are associated with the development of automatic habits, persisting even in the face of adverse changes (42, 43). Therefore, we hypothesize that glutamate receptors in the dorsal striatum may influence nicotine addiction by altering various glutamate neurotransmitters.

### Limitations

This study has several limitations. (1) The study subjects were all Han males, so the results are not universally applicable to the population. (2) Isolated lesions of the dorsal striatum are infrequent in clinical practice, and despite extensive efforts, the sample size remains modest. We aim to incorporate additional data in subsequent studies to corroborate and expand our conclusions. (3) Our control group consisted of individuals with damage in other areas of the brain that may include areas potentially involved in the nicotine addiction pathway (as the mechanism of nicotine addiction is currently unclear), which may affect the accuracy of our observations. (4) The precise mechanisms by which the dorsal striatum contributes to nicotine dependence remain conjectural. Although preliminary animal experiments have been conducted and it has been found that the expression of GABA_A_ α1 protein in the dorsal striatum of nicotine addicted mice is downregulated, our experimental progress has been limited due to the high mortality rate of mice with dorsal striatum injury. I hope more people will participate in this experiment in the future.

## Conclusion

Our research results observed that patients with dorsal striatum damage are more prone to smoking addiction interruption, which occurs earlier and more easily, and that patients with more severe damage may maintain this interruption for a longer duration. Additionally, patients with dorsal striatum damage have relatively lower FTND scores, indicating a lower degree of nicotine dependence.

## Data Availability

The original contributions presented in the study are included in the article/supplementary material, further inquiries can be directed to the corresponding author.
